# Combined AFM and super-resolution localisation microscopy: Investigating the structure and dynamics of podosomes

**DOI:** 10.1016/j.ejcb.2020.151106

**Published:** 2020-09

**Authors:** Liisa M. Hirvonen, Richard J. Marsh, Gareth E. Jones, Susan Cox

**Affiliations:** Randall Centre for Cell and Molecular Biophysics, King's College London, Guy's Campus, London SE1 1UL, UK

**Keywords:** AFM, atomic force microscopy, ECM, extracellular matrix, HAWK, Haar Wavelet Kernel analysis, PALM, photoactivated localisation microscopy, SIM, structured illumination microscopy, SMLM, single molecule localisation microscopy, STORM, stochastic optical reconstruction microscopy, Podosome, Localisation microscopy, Multi-modal microscopy, Super-resolution, AFM

## Abstract

Podosomes are mechanosensitive attachment/invasion structures that form on the matrix-adhesion interface of cells and protrude into the extracellular matrix to probe and remodel. Despite their central role in many cellular processes, their exact molecular structure and function remain only partially understood. We review recent progress in molecular scale imaging of podosome architecture, including our newly developed localisation microscopy technique termed HAWK which enables artefact-free live-cell super-resolution microscopy of podosome ring proteins, and report new results on combining fluorescence localisation microscopy (STORM/PALM) and atomic force microscopy (AFM) on one setup, where localisation microscopy provides the location and dynamics of fluorescently labelled podosome components, while the spatial variation of stiffness is mapped with AFM. For two-colour localisation microscopy we combine iFluor-647, which has previously been shown to eliminate the need to change buffer between imaging modes, with the photoswitchable protein mEOS3.2, which also enables live cell imaging.

## 1 Introduction

Podosomes are highly dynamic micron-sized conical integrin-based adhesion structures that form on the surface of certain cells, especially those of monocytic origin. They are mechanosensitive, i.e. they can sense the extracellular matrix (ECM) topography and rigidity, and besides adhesion they are capable of remodelling and digesting the ECM. They are pivotal in many cellular processes that require matrix remodelling, such as the spread of cancer cells and inflammatory responses of macrophages, bone resorption by osteoclasts, and remodeling of blood vessels and axons by endothelial cells. Podosomes are linked to diseases such as cancer metastasis, chronic inflammations, cardiomyopathy and age-related osteoporosis (Maridonneau-Parini (2014), Paterson and Courtneidge (2018)). Understanding how and why podosomes form, and the molecular mechanics behind how they function, will play a central role in the prevention and treatment of these diseases.

Podosomes have been studied extensively by conventional light microscopy (Marchisio et al. (1984), Pfaff and Jurdic (2001), Destaing et al. (2003)), electron microscopy (Luxenburg et al. (2007), Gawden-Bone et al. (2010), Schmidt et al. (2011), Joosten et al. (2018)) and atomic force microscopy (Labernadie et al., 2010, Labernadie et al. (2010, 2014). These studies have yielded a model of the podosome, consisting of an actin-rich core nucleated by the ARP2/3 complex, surrounded by a ring of adhesion-related proteins such as talin, vinculin, paxillin and zyxin, which together with integrins anchor the podosome onto the ECM and provide structural support during force generation. Podosomes generate forces to protrude into the ECM to probe the matrix topography and stiffness, but the mechanism behind this force generation is unclear. Podosomes are also capable of digesting and remodelling the ECM (Paterson and Courtneidge (2018)), but little is known about the molecular mechanism behind these processes.

Live cell imaging is essential in mechanobiological investigations which study function and mechanics, and it would be helpful to have many forms of dynamic information on a small spatial scale – ideally small enough to resolve single molecules. Atomic force microscopy (AFM) (Binnig et al. (1986)) and fluorescence microscopy are a powerful combination in providing different types of information that complement each other (Hinterdorfer and Dufrêne (2006), Müller and Dufrêne (2008)), and both are compatible with physiological buffers, allowing the observation of living biological specimens. Fluorescence microscopy allows the tagging of intracellular molecules and cellular components with high specificity, and their observation inside cells in a minimally invasive manner using non-destructive wavelengths of light in the visible spectrum. AFM, on the other hand, uses a sharp tip to measure the topography of the sample with sub-nanometer axial resolution, or other physical properties, such as adhesion or stiffness. It is also possible to functionalise AFM tips to recognise specific molecules and measure binding energies (Hinterdorfer and Dufrêne (2006), Miller et al. (2017)), or use AFM for manipulation of the sample in nanometer scale (Chacko et al. (2013b)).

In the past, combination of fluorescence microscopy and AFM was made difficult by the diffraction limit of light microscopy, which restricted the resolution in fluorescence microscopy to two orders of magnitude more than AFM. Recently developed super-resolution microscopy techniques are able to go beyond this limit, but the achievable resolution depends on the technique. Currently, structured illumination microscopy (SIM) is the most utilised super-resolution method for live cell imaging due to its speed. The combination of SIM and AFM has been demonstrated (Gomez-Varela et al., 2020),(and SIM has been applied to study podosomes (Cervero et al., 2018; Rafiq et al., 2019a; Rafiq et al., 2019; van den Dries et al., 2019). However, as SIM has only a maximum of 2-fold potential for resolution improvement over conventional fluorescence microscopy and a typical final resolution around 120–140 nm, its usefulness in molecular mechanobiology is very limited.

Fluorescence super-resolution techniques based on single molecule localisation microscopy (SMLM), such as stochastic optical reconstruction microscopy (STORM) (Rust et al. (2006)) and photoactivated localisation microscopy (PALM) (Betzig et al. (2006)), offer theoretically unlimited resolution which is in practise limited by the signal-to-noise ratio typically to few tens of nanometres, a similar scale to the typical lateral resolution of AFM when imaging soft biological samples (Miller et al. (2017), Hauser et al. (2017), Zhou et al. (2017), Chacko et al. (2013a)). Localisation microscopy methods have been applied to the study of podosomes with the aim of resolving the molecular architecture. While fixed cell SMLM imaging can shed light on the molecular architecture of podosomes (Bouissou et al. (2017), Joosten et al. (2018), Foxall et al. (2019)), many studies have also yielded conflicting results due to artefacts arising from sample preparation and image processing protocols (Cox et al. (2012), van den Dries et al. (2013), Meddens et al. (2014), Walde et al. (2014), Staszowska et al. (2017)).

Localisation microscopy techniques rely on the ability to switch a fluorophore between a bright and a dark state. In its most simple experimental form, direct STORM (dSTORM) (Heilemann et al. (2008), van de Linde et al. (2011)), the sample is illuminated with a high power laser while immersed in a switching buffer that makes the dye molecules blink, and a series of images is acquired where each frame contains only a few emitting fluorophores. While the images of the fluorophores are diffraction-limited, their centroid positions can be calculated with great accuracy, and the final image constructed by summing these locations together. However, to induce the blinking, a switching buffer containing enzymatic oxygen scavengers is normally required. Unfortunately the typical ingredients of this buffer stick to the AFM cantilevers, and make AFM image acquisition impossible. Most attempts to combine AFM and STORM report adding the switching buffer for STORM after AFM imaging (Odermatt et al. (2015), Bondia et al., 2017a, Bondia et al. (2017a, b), Monserrate et al. (2014)), which is time-consuming and cumbersome and can lead to sample movement and damage, seriously limiting the practical use of this technique. In this work, we perform STORM imaging with the fluorescent dye iFluor-647, which is especially suitable for imaging in a buffer without enzymatic oxygen scavenger (Hirvonen and Cox (2018)), allowing combined AFM and STORM imaging without any change of buffer.

The use of endogenous fluorescent proteins, such as the green fluorescent protein (GFP), offers an alternative to antibody labelling. With some fluorescent proteins – called photoswitchable fluorescent proteins – the on and off states can be controlled with specific wavelengths of light. Photoswitchable fluorescent proteins offer another way to perform localisation microscopy without special buffers, and while they also enable live cell imaging, the cost of better resolution in SMLM is increased acquisition time: typically thousands of images are required for the reconstruction of the final image, and the acquisition of this number of frames usually takes at least several minutes – too slow for live cell imaging. Acquisition speed can be increased by acquiring data with higher emitter density (i.e. more molecules/frame), but any overlap of molecules in the raw data can lead to artefacts in the reconstructed final image, such as artificial sharpening of sample structures and missing features. It can be very difficult to spot these artefacts, since there is no warning of failure by the reconstruction software, and quantification of resolution, for example by Fourier Ring Correlation (FRC) (Nieuwenhuizen et al. (2013)), indicates sharpened images have a better resolution.

Artificial sharpening has been shown to be a problem with most localisation reconstruction software where the overlapping molecules cannot be separated properly (Burgert et al. (2015), Sage et al. (2015), Fox-Roberts et al. (2017)), even methods designed for high density SMLM data. To address this problem, we have recently developed a new method, Haar Wavelet Kernel (HAWK) analysis (Marsh et al. (2018)), for separating overlapping molecules. HAWK is a preprocessing method that separates molecules by their blinking statistics, and has been shown to eliminate sharpening artefacts in high density localisation microscopy data. HAWK also works for extremely high density data, allowing reconstruction of a final image from just a few hundred frames, and bringing data acquisition time down to a few seconds – fast enough for many live cell imaging applications.

In this work we have labelled several podosome-related proteins with the photoconvertible fluorescent protein mEOS3.2 (Zhang et al. (2012)), which usually emits in green with 488 nm excitation but can convert to yellow-emitting conformation upon 405 nm illumination. We use mEOS3.2 and iFluor-647 labelling of podosome molecular components for combined two-colour localisation microscopy and AFM on a single microscope, where localisation microscopy provides resolution of <30 nm, while the spatial variation of stiffness is mapped with AFM. We also demonstrate typical artefacts created by super-resolution imaging, and how these can be reduced by HAWK imaging.

## 2 Methods

### 2.1 Sample preparation

mEOS3.2 sequence was amplified from a template (gift from Dylan Owen, King's College London) using PCR and cloned into an pLNT/Sffv-MCS vector via pCR Blunt vector (Invitrogen). cDNAs encoding the target sequences: residues 1975–2541 of human talin from a template plasmid we previously generated (Vijayakumar et al. (2015)), paxillin and lifeact (gifts from Maddy Parsons, King's College London), and ARPC3 (gift from Matthias Krause, King's College London), were amplified using PCR and then cloned via pCR Blunt vector into the multiple cloning site of the pLNT/Sffv-mEOS3.2-MCS vector. VSV-G pseudotyped lentiviruses encoding mEOS3.2-talin (1975-2541), mEOS3.2-paxillin, mEOS3.2-lifeact or mEOS3.2-ARPC3 were packaged in HEK-293T cells by transient transfection of the cells with the *p*Δ8.91 and pMD.G accessory plasmids along with the pLNT/Sffv transfer vector. Supernatants containing lentivirus were harvested after 48 h and filtered through a 0.45 μm filter. THP-1 cells (ATCC #TIB-202TM) were incubated with lentiviral supernatants in 12-well plates for 72 h and washed thoroughly. The cultures were then expanded in 37 °C, 5% CO_2_ in RPMI-1640 medium (R0883, Sigma) supplemented with 10% heat-inactivated FBS (SV30160.03, GE Healthcare), 1% penicillin/streptomycin (P0781, Sigma), and 0.05 mM *β*-mercaptoethanol (125472500, ACROS). For imaging, 35 mm dishes with #1.5 glass coverslip bottom (WPI, FL) were coated with 10–15 μl/ml bovine fibronectin (F1141, Sigma) in PBS for 24 h at 37 °C. After 2× wash with PBS, 6 × 10^5^ cells were seeded on each dish with 2 ng/ml recombinant human TGF-*β* (240-B, R&D Systems).

For fixed cell experiments, the cells were incubated in 37 °C, 5% CO_2_ for 24 h. If the cells were unroofed, the medium was replaced with H_2_O buffer containing 10 μg/ml phalloidin (sc-202763, Santa Cruz Biotechnology) and protease inhibitors (04693124001, Roche) for 40 s, and the cells were then flushed 10× with the same buffer. Both unroofed and intact cells were fixed for 20 min with 4% formaldehyde, and non-unroofed cells were permeabilised for 5 min in 0.1% Triton X-100. For actin staining, iFluor-647-phalloidin (23127, AAT Bioquest, CA) stock solution was diluted 1:500 in 3% BSA in PBS, and the cells incubated for 30 min in the dye solution. For vinculin staining, the cells were blocked for 30 min in 3% BSA in PBS, incubated for 1 h with anti-vinculin mouse antibody (V9131, Sigma) diluted 1:200 in 3% BSA in PBS, washed thoroughly, and incubated for 1 h with anti-mouse iFluor-647 (16783, AAT Bioquest) diluted 1:500 in 3% BSA in PBS. For imaging, MEA stock solution (1 M cysteamine (30070, Sigma–Aldrich) in H_2_O, pH adjusted to 8.0 with HCl solution) was diluted in TN buffer (H_2_O with 50 mM Tris pH 8.0 and 10 mM NaCl) at a final concentration of 50 mM immediately before imaging, and added to the sample dish before imaging.

For live cell experiments the cells were incubated in culture medium at 37 °C, 5% CO_2_ for 24–48 h after seeding, and the medium was then changed to RPMI-1640 without phenol red (R7509, Sigma) supplemented with 10% FBS before imaging.

### 2.2 Data acquisition

**Fig. 1:**
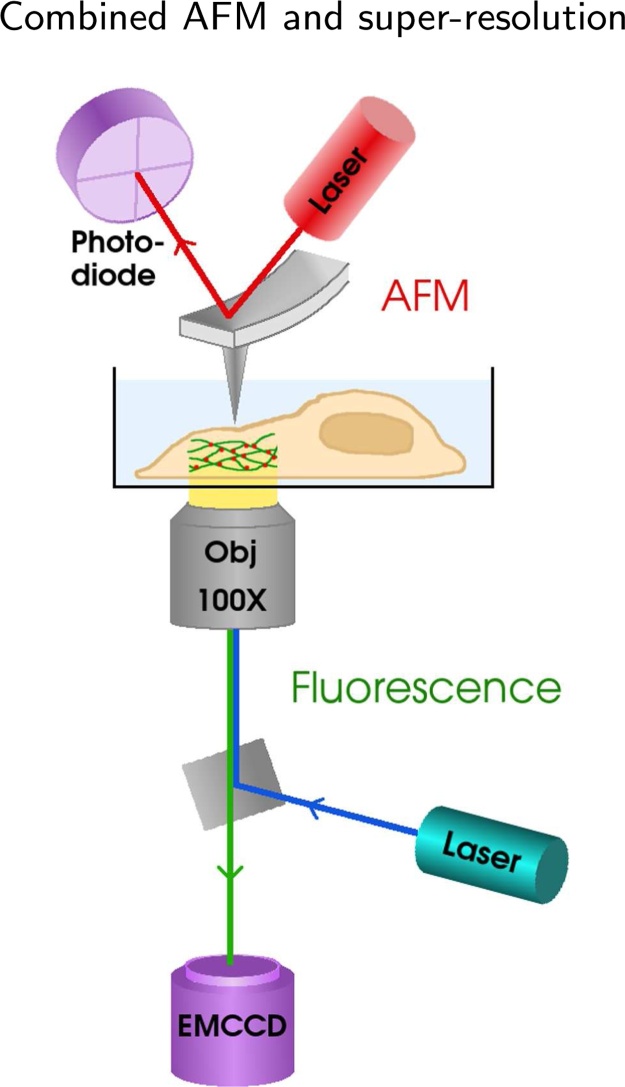
A simplified schematic diagram of the combined AFM + STORM microscope setup. The setup was built around a standard inverted microscope base, with an AFM to image the sample from top, and a fluorescence setup underneath for STORM imaging. AFM scans a sharp tip over the sample surface and produces a topographic image of the sample, while in fluorescence microscopy cellular components are labelled with fluorescent tags which light up under illumination with specific wavelengths, allowing imaging inside cells.

Samples were imaged with our custom-built hybrid system combining AFM and fluorescence imaging; see Fig. 1 for a schematic diagram. The setup was built around a standard inverted microscope base (Zeiss Axio Observer.Z1) and equipped with a LightHUB-6 laser combiner (Omicron, Germany) with 405 nm, 488 nm, 647 nm (Omicron, Germany) and 561 nm (Cobolt, Sweden) lasers for fluorescence excitation, an EMCCD (Andor iXon Ultra) for fluorescence data collection, and a JPK Nanowizard 3 for AFM imaging. For STORM/PALM, the sample was illuminated and imaged from the bottom through a 100× NA 1.4 oil immersion objective (Zeiss Plan-Apochromat). For fixed cell localisation microscopy a total of 15,000–30,000 frames were acquired. The instrumentation for fluorescence data collection was controlled with *μ*Manager software (Edelstein et al. (2014)). HAWK imaging was performed on a setup described previously (Marsh et al. (2018)), using similar settings.

For AFM imaging, a SiN cantilever with a Si tip with nominal spring constant of 0.081 N/m, tip radius <10 nm and gold coating on the reflex side (HYDRA-6V-200WG, Applied NanoStructures, CA) was mounted on the AFM head, and the head was placed on the top of the sample. Images were recorded on quantitative imaging (QI™) mode, which records a complete force-distance curve for each pixel without exerting lateral forces on the sample. The set point was 8 nN with 1000 nm ramp size and 15 ms pixel time. The scan times for the AFM images in Figs. 3(b,c) and 3 (e,f) were 37 and 16 min, respectively. The AFM images were processed by subtracting a 1st degree polynomial fit from each line.

### 2.3 STORM/PALM data processing

The raw localisation microscopy images were processed with ThunderSTORM (Ovesný et al. (2014)) software using default processing parameters and final pixel size of 10 nm. The reconstructed images were then post-processed to only select molecules with 50 nm <*σ* < 250 nm where *σ* is related to the size of the spot, and molecules appearing in consecutive frames were merged with merging radius of 20 nm and maximum 1 off-frame between detections. Gaussian blur was added to the final images to reduce noise.

For live cell localisation microscopy, the stacks containing high density data were pre-processed with HAWK software (Marsh et al. (2018)) to separate overlapping molecules. The derived data stacks were then processed with ThunderSTORM using default processing parameters, and post-processed to only select molecules with 70 nm <*σ* < 250 nm.

## 3 Results

### 3.1 Two-colour localisation microscopy

To test two-colour localisation microscopy in a buffer that is also compatible with AFM imaging, THP-1 cells expressing mEOS3.2 constructs were plated onto coverslip-bottom dishes and induced to form podosomes (Bombara and Ignotz (1992), Rafiq et al. (2017)), then fixed and immunolabelled with iFluor-647, and imaged in the MEA buffer. The 647 channel was acquired first, and the photoconverted form of mEOS3.2 was then imaged in the 561 channel.

**Fig. 2:**
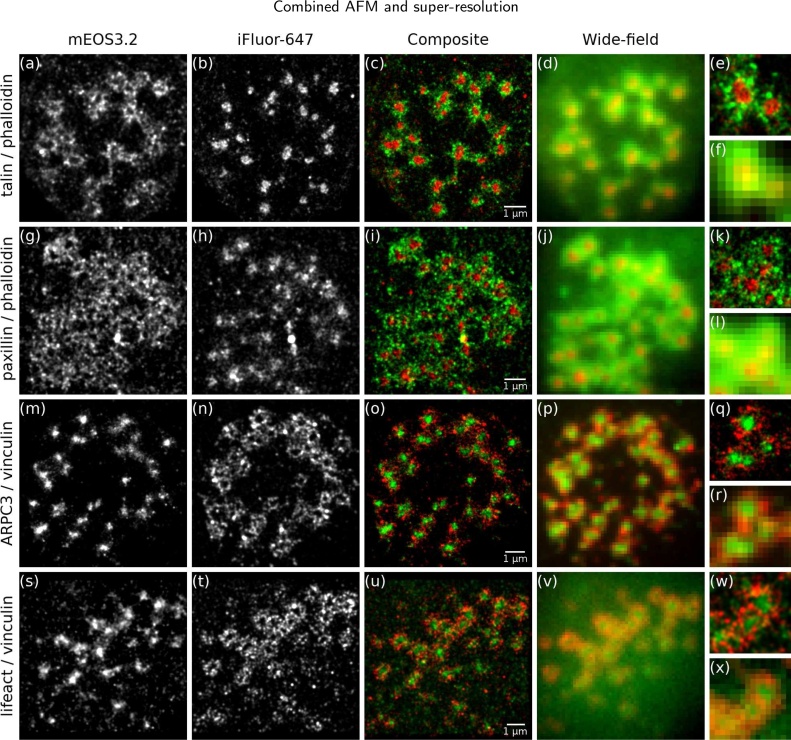
Two-colour localisation microscopy (columns 1–3) of podosome molecular components labelled with mEOS3.2 (1st column, and green in composite images) and iFluor-647 (2nd column, and red in composite images) significantly improves resolution over wide-field imaging (4th column). mEOS3.2-tagged talin (1st row) and paxillin (2nd row) localise to the ring with iFluor-phalloidin highlighting the actin-rich core, while mEOS3.2-tagged ARPC3 (3rd row) and lifeact (4th row) localise to the core and iFluor-labelled vinculin forms a ring. The last column shows enlarged areas of the composite localisation microscopy (top) and wide-field (bottom) images. (For interpretation of the references to color in this figure legend, the reader is referred to the web version of this article.)

Fig. 2 shows localisation microscopy images of podosomes with various molecular components labelled with mEOS3.2 (green) and iFluor-647 (red). As expected, talin (Fig. 2a–df) and paxillin (Fig. 2g–l) localise to the podosome ring; here the core is labelled with iFluor-647-phalloidin. ARPC3 (Fig. 2m–r) and lifeact (Fig. 2s–x) localise to the core, as expected, and vinculin labelled with iFluor-647 shows the podosome ring. In Fig. 2 localisation microscopy (columns 1-3) clearly improves the resolution over conventional wide-field microscopy (column 4). The localisation uncertainty was calculated as 23.2 ± 1.6 nm for mEOS3.2 and 26.1 ± 7.5 nm for iFluor, with an average of 900 ± 267 photons detected from each mEOS3.2 molecule and 782 ± 179 photons from each iFluor dye molecule, and estimated resolution of the super-resolution images <30 nm.

### 3.2 Combined AFM and localisation microscopy

**Fig. 3:**
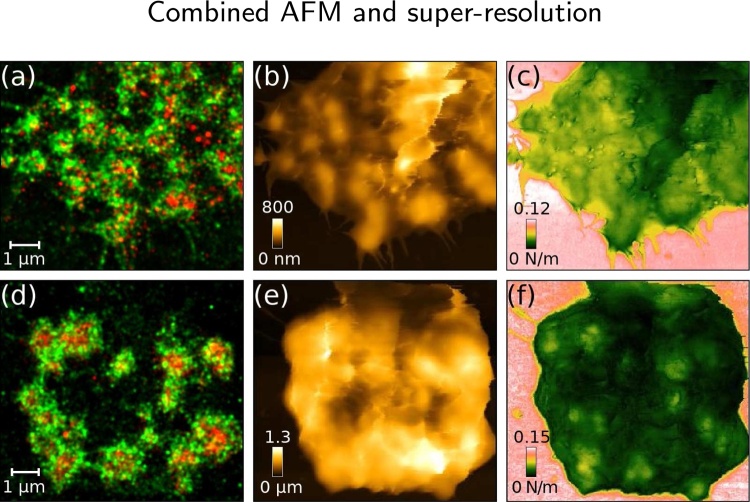
Combined AFM and STORM images of podosomes in (a–c) unroofed and (d–f) intact fixed THP-1 cells. (a,d) Localisation microscopy images of mEOS3.2-tagged talin (green) and iFluor-phalloidin (red). In the unroofed cell, the podosome cores can be seen as higher areas in the AFM (b) height and (c) stiffness images. In the intact cell the podosomes are difficult to see in the AFM (e) height image due to a fluffy membrane, but the (f) stiffness image clearly shows the podosome cores as stiffer areas under the membrane. (For interpretation of the references to color in this figure legend, the reader is referred to the web version of this article.)

Fig. 3shows an example of podosomes imaged with combined two-colour localisation microscopy and AFM. The localisation microscopy images (Fig. 3a,d) show the actin-rich podosome cores labelled with iFluor-phalloidin (red) surrounded by a mEOS3.2-tagged talin ring (green). The iFluor-647 (red) localisation image was acquired first, then the mEOS3.2 (green) localisation image, and the AFM scan directly afterwards without buffer change, as simultaneous acquisition is not practical due to the overlapping spectrum of the imaging and AFM laser wavelengths.

While the localisation microscopy images of the two cells look similar with the iFluor-phalloidin labelled core surrounded by the mEOS3.2-labelled talin ring (Fig. 3a,d), differences can be seen in the AFM images. In the unroofed cell, where the top membrane was removed prior to imaging, the AFM height (Fig. 3b) and stiffness (Fig. 3c) images show the podosomes as features higher and stiffer than the area immediately surrounding them, as expected from the high concentration of f-actin in the podosome core. In the intact cell, the podosomes are difficult to see in the AFM height image (Fig. 3e) due to the membrane on top of the podosomes. However, the AFM stiffness image (Fig. 3f) clearly shows the podosome cores as stiffer areas under the membrane, suggesting that it will be possible to perform live cell podosome stiffness measurements through the membrane.

### 3.3 HAWK imaging

**Fig. 4:**
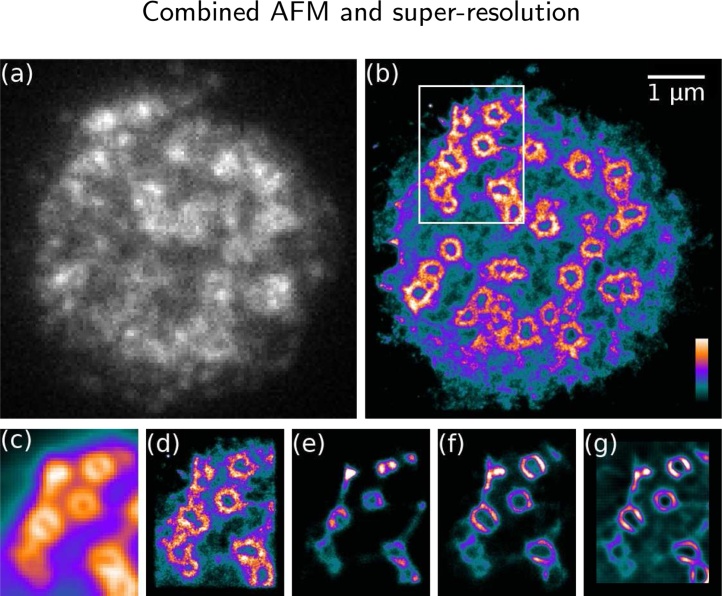
Live cell localisation microscopy images of mEOS3.2-labelled talin in podosomes in a THP-1 cell. (a) A frame of raw data showing high density of emitters, and (b) HAWK-processed final image. (c–g) Comparison of (c) wide-field image and different super-resolution processing methods: (d) HAWK, (e) single-emitter fitting, (f) multi-emitter fitting, and (g) SRRF. While HAWK shows the expected podosome ring structure, other methods show artefacts including sharpening, contraction of the ring and missing features. Colour bar in (b) indicates intensity scale. (For interpretation of the references to color in this figure legend, the reader is referred to the web version of this article.)

Live cell localisation microscopy was performed with THP-1 cells transfected with the mEOS3.2-talin construct. Data was acquired at high fluorophore density; an example frame from the data set is shown in Fig. 4a, revealing high overlap of fluorescent molecules. HAWK processing of this data (Fig. 4b,d) produces an image that shows the expected podosome ring structure and a lower concentration of talin distributed throughout the cell. Fig. 4c–g shows comparison of different processing methods in the small area indicated in Fig. 4b. The general podosome ring structure can be seen in the wide-field image (Fig. 4c) but the diffraction-limited resolution obscures all other details seen in the HAWK processed super-resolution image (Fig. 4d). Traditional single-emitter fitting (Fig. 4e) produces very strong artefacts related to high molecule density in the raw data, e.g. artificial sharpening, contraction of the ring structures and missing features. Methods designed for high density data, multi-emitter fitting (Fig. 4f) and SRRF (Gustafsson et al. (2016)) (Fig. 4g), improve the image compared to single-emitter fitting but still show strong sharpening artefacts and missing information.

## 4 Discussion

In a quest to elucidate the mechanobiology behind podosome structure and function, a variety of different kinds of information is required. Advances in super-resolution microscopy methods have significantly improved the scale where we can make direct observations of the distribution of fluorescently labelled biomolecules inside cells from hundreds of nanometers to near molecular scale. However, there are drawbacks.

Firstly, all microscopy techniques are prone to artefacts. This is especially true for super-resolution techniques which are relatively new and often designed for specific types of data. Artefacts can be created by the fluorescence labelling process (Whelan and Bell (2015)), the data acquisition process, the image processing method, or a combination of these. For example, if the labelling density is not high enough or the image acquisition time is too short, continuous structures may appear discontinuous. On the other hand, too high a density of fluorescent labels in SMLM data can lead to artificial sharpening of sample structures. Another common problem with immunofluorescence labelling is the binding of multiple secondary antibodies to a primary antibody (Maidorn et al. (2016)), which is not visible in conventional fluorescence imaging but leads to artificial clustering of single molecules in SMLM imaging. An image with artefacts no longer gives an accurate representation of the sample structure, and is open to misinterpretation.

Secondly, while the observation of living cells is essential for obtaining functional and dynamic information, there is usually a trade-off between resolution and speed. There is certainly a need for live-cell super-resolution imaging, and super-resolution methods based on structured illumination have become increasing popular in filling this gap, but their potential for resolution improvement is limited to a factor of 2, leading to a final resolution still over 100 nm. SMLM techniques, on the other hand, can reach single molecule scale resolution but at the cost of increased acquisition time. Many SMLM processing methods have tried to address this problem and are indeed capable of constructing an image from high density SMLM data which can be acquired faster, but often with severe artefacts in the final image. Our new technique HAWK, which is able to process extremely high density SMLM without artificial sharpening, opens up new avenues for live-cell super-resolution microscopy with improved resolution in the scale of few 10 s of nm and data acquisition time of a few seconds. This time scale is ideal, for example, for the study of podosome dynamics in living cells.

Finally, combining super-resolution microscopy with other methods can yield different types of information, such as mechanical property maps, in addition to the location of the labelled biomolecules. Recent advances in both super-resolution microscopy and AFM have made a combination of these techniques a desirable tool especially for nanoscale mechanobiological research. A major drawback in combining AFM with localisation microscopy has been that the standard STORM buffer, containing enzymatic oxygen scavenger, is not compatible with AFM cantilevers, and the combination of STORM and AFM has usually required a buffer change between the imaging modalities (Odermatt et al. (2015), Bondia et al., 2017a, Bondia et al. (2017a, b), Monserrate et al. (2014)), which is cumbersome and leads to longer time intervals and possible movement and damage to the sample between the images. In this work, we have used a new red cyanine dye, iFluor-647, for combined AFM and STORM imaging of podosomes in THP-1 cells in a buffer without enzymatic oxygen scavenger. This buffer is compatible with AFM imaging such that no buffer change is required between the imaging modalities, simplifying the process and eliminating artefacts in correlative AFM and STORM imaging.

The field of microscopy is developing at a fast pace. Super-resolution microscopy methods which were first demonstrated less than 15 years are now becoming everyday instruments in biological sciences, and the methods for perfecting image reconstruction to faithfully represent the sample structure are still being developed. AFM is now routinely used for imaging living biological samples, and increasingly combined with super-resolution imaging methods. The combination of live-cell AFM with HAWK super-resolution imaging is an exciting development which will allow simultaneous measurement of molecular architecture and mechanical properties, and will be useful in understanding podosome structure and function.

## 5 Conclusion

In this work we demonstrate an easy and straightforward method for combined AFM and SMLM super-resolution imaging of podosomes. While the combination of these methods has previously required buffer change between the imaging modes, this problem is eliminated using iFluor-647 dye in a simple buffer without an enzymatic oxygen scavenger. The use of endogenous fluorescent proteins offers an alternative to antibody labelling, and enables live-cell imaging which can be difficult with dye labels. We have combined mEOS3.2 with iFluor-647 for labelling podosome molecular components in fixed THP-1 cells, and demonstrated two-colour localisation microscopy with AFM imaging in one setup. We also demonstrate live-cell localisation microscopy of podosomes using a new localisation microscopy method HAWK, which speeds up the localisation microscopy data collection time from 10 s of minutes to a few seconds.

Besides podosomes, the methods introduced here can be applied to the study of a wide range of biophysical processes in any cell biology studies, while HAWK will advance a wide variety of biological imaging applications where the speed of data acquisition is a critical parameter and resolution below the diffraction limit is needed.

## Conflicts of interest

The authors declare no competing interests.
